# Research on the Response Characteristics of Vanadium Pentoxide Film to the Irradiation of Ultrafast Pulsed Laser

**DOI:** 10.3390/nano11082078

**Published:** 2021-08-16

**Authors:** Qianqian Shi, Guodong Zhang, Yuheng Wang, Yu Lan, Jiang Wang, Guanghua Cheng

**Affiliations:** 1 State Key Laboratory of Transient Optics and Photonics, Xi’an Institute of Optics and Precision Mechanics, Chinese Academy of Sciences, Xi’an 710119, China; shiqianqian@opt.cn (Q.S.); lanyu2017@opt.cn (Y.L.); 2 University of Chinese Academy of Sciences, Beijing 100049, China; 3 School of Artificial Intelligence, Optics and Electronics (iOPEN), Northwestern Polytechnical University, Xi’an 710072, China; guodongzhang@nwpu.edu.cn (G.Z.); wjiang@nwpu.edu.cn (J.W.); 4 Research Center of Semiconductor Lighting and Information Engineering Technology, South China University of Technology, Guangzhou 510641, China; imtree@126.com

**Keywords:** vanadium pentoxide, ultrafast laser irradiation, Z-scan technique, nonlinear optical

## Abstract

Vanadium pentoxide (V_2_O_5_) is the most stable phase among many transition metal vanadium oxides, and has already been widely used in many fields. In this study, the morphological, structural, and optical responses of V_2_O_5_ film to ultrafast laser irradiation was investigated. The third-order nonlinear optical properties of V_2_O_5_ film were measured by common Z-scan technique, and the results showed that V_2_O_5_ film has self-defocusing and saturable absorption characteristics. The third-order nonlinear absorption coefficient and nonlinear refractive index were calculated to be −338 cm/GW and −3.62 × 10^−12^ cm^2^/W, respectively. The tunable saturated absorption with modulation depth ranging from 13.8% to 29.3% was realized through controlling the thickness of vanadium pentoxide film. V_2_O_5_ film was irradiated by ultrafast laser with variable pulse energy, and the morphological and structural responses of the V_2_O_5_ to the laser with different energy densities were investigated. The irreversible morphological and structural responses of V_2_O_5_ films to ultrafast laser irradiation was analyzed using the phase-contrast microscope and Raman spectrum. The chemical structure change from V_2_O_5_ to V_6_O_13_ was considered the main reason for refractive index modification.

## 1. Introduction

Vanadium oxides are known to have the unique property of semiconductor–metal reversible phase transitions during long-pulsed laser irradiation, thus having a wide range of potential applications in optical, electronic, and photoelectric devices [1]. There are more than ten stoichiometric formulae for vanadium oxides from the lowest VO phase to the highest V_2_O_5_ phase [2]. Since the vanadium atom in V_2_O_5_ has the highest valence, V_2_O_5_ possesses the most stable chemical properties among these vanadium oxides. V_2_O_5_ has a reversible first-order phase transition property, and its phase transition temperature is about 257 °C [3]. In addition, V_2_O_5_ has a unique layered structure, which makes it a very promising material.

Compared to bulk materials, nanofilms exhibit many excellent electrical and optical properties such as high thermal conductivity, high light transmittance, outstanding nonlinear optical properties, etc. [4]. Therefore, the best utilization and application form of V_2_O_5_ is the two-dimensional film. At present, there are many techniques for preparing thin film, such as magnetron sputtering, Sol-gel, atom layered deposition (ALD), and hydrothermal [5,6,7,8]. The photoelectric properties of thin films are slightly different due to the different preparation techniques and technological parameters. In our experiment, silica coating was used to improve the stability of the V_2_O_5_ film [9].

With the appearance and flourishing of the ultrafast laser, more researchers are now involved in the study of the interaction between ultrafast laser and material [10,11,12]. Due to very high peak power at focal region, the advantage of nonlinear optical absorption and localized energy deposition has conferred a unique potential upon ultrafast laser pulses to structure and modify optical material. Some transition metal oxides possess excellent nonlinear optical properties, which can be controlled by changing the samples’ shape and thickness, such as VO*x*, WS_2_, and MoS_2_ [13,14,15]. Although there are several works related to the optical and electrical properties of V_2_O_5_ thin films [16,17,18], few studies have been conducted on the performance response of V_2_O_5_ induced by ultrafast laser.

In this work, the response characteristics of V_2_O_5_ film to the irradiation of ultrafast pulsed laser is carefully studied. V_2_O_5_ films with different thicknesses were prepared by radio frequency (RF) magnetron sputtering technology. The Z-scan setup equipped with the Yb-doped fiber Chirped Pulsed Amplification (CPA) laser system was used to characterize the nonlinear optical properties of the V_2_O_5_ film. In addition, the laser, with different pulse energies, is used to irradiate the V_2_O_5_ film, which is a supplementary process to the Z-scan experiment. For analyzing the morphological and structural responses of V_2_O_5_ film to the ultrafast laser irradiation, Scanning Electron Microscopy, Phase-Contrast Microscopy, and the Raman spectrum were utilized in the experiment.

## 2. Materials and Methods

### 2.1. Film Preparation

The radio frequency (RF) magnetron sputtering was used to deposite vanadium pentoxide films on the 1 × 1 cm^2^ R-plane double-sided polished sapphire substrates. The temperature for film deposition was set as 395 °C and the radio frequency power was kept at 200 W. The working pressure was kept at 0.7 Pa, while the Ar and O_2_ flow were set at 98 sccm and 2.1 sccm, respectively. By controlling the deposition time, the vanadium oxide films with different thicknesses were prepared. After deposition, samples were annealed at 395 °C in situ in the Ar-filled chamber for 40 min. In order to prevent the films from being oxidized, a 24 nm thick silicon oxide protective film was coated onto the sample surface.

### 2.2. Picosecond Laser Processing

The Yb-doped fiber Chirped Pulsed Amplification (CPA) laser system, operating at a wavelength of 515 nm with pulse duration of 1.2 ps and repetition rate of 100 KHz, was used to irradiate V_2_O_5_ thin films under different pulse energies. Through an objective lens (Mitutoyo), with numerical aperture (NA) of 0.42, the ultrafast laser was focused onto the vanadium oxide films, which were placed on a three-dimensional stage (Aerotech ANT95). Then, the ultrafast laser pulses with different pulse energies were used to selectively modify spaces on the vanadium oxide films in a single shot regime. The separation distance between adjacent laser-modified regions was set as 10 μm in the experiment.

### 2.3. Characterization

The surface morphology of the V_2_O_5_ thin film and the laser-induced modification were studied by Scanning Electron Microscopy (SEM, FEI, Verios G4, Hillsboro, Oregon, USA), Atom Force Microscopy (AFM, Bruker, Dimension Icon, Mannheim, Germany), X-ray photoelectron spectroscopy (XPS, Shimadzu, Kratos Ultra DLD, Kyoto, Japan), and Phase-Contrast Microscopy (Olympus, BX51, Tokyo, Japan). The UV-Vis-NIR transmittance spectrum was measured by the spectrophotometer (Shimadzu, UV-3150, Kyoto, Japan) at room temperature. The modifications in chemical structure induced under different pulse energies were characterized using the Confocal Micro-Raman spectrometer (WITec, Alpha300R, Ulm, Germany) with excitation laser of 532 nm. We expected to see a larger nonlinear effect at longer wavelength; therefore, the Z-scan setup equipped with the Yb-doped fiber Chirped Pulsed Amplification (CPA) laser system operating at a wavelength of 1030 nm was used to characterize the nonlinear optical properties of the V_2_O_5_ thin film.

## 3. Results and Discussion

### 3.1. Surface Morphology and Component

**Figure 1:**
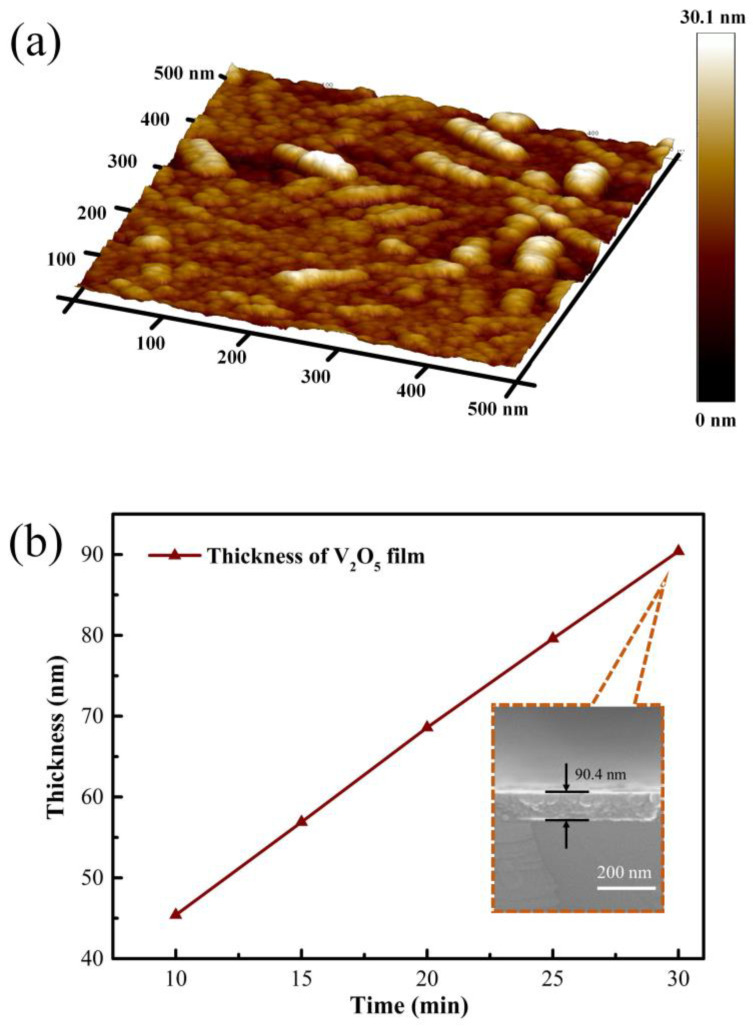
Three-dimensional morphology of V_2_O_5_ thin film on R-plane sapphire substrate (**a**), the synthesized V_2_O_5_ film with different thickness and SEM cross section image (**b**).

The surface morphology of the synthesized V_2_O_5_ thin film was characterized using the AFM, as shown in Figure 1a. It can be seen from the three-dimensional AFM image that the prepared V_2_O_5_ thin film on R-plane sapphire substrate possesses a superior nanorod microcrystalline structure composed of smaller nanoparticles. The roughness of the film prepared was 2.64 nm. For controlling the thickness of the V_2_O_5_ film, the sputtering time during the material deposition process was regulated, ranging from 10 min to 30 min. The thickness of the V_2_O_5_ film was characterized by the SEM and step profiler. The results are concluded as shown in Figure 1b; the thickness of these five films were 45.4 nm, 56.9 nm, 68.6 nm, 79.6 nm, and 90.4 nm, respectively. It can be derived that the increase of film thickness was positively linear with the sputtering time. The deposition rate was about 2.25 nm/min. 

**Figure 2:**
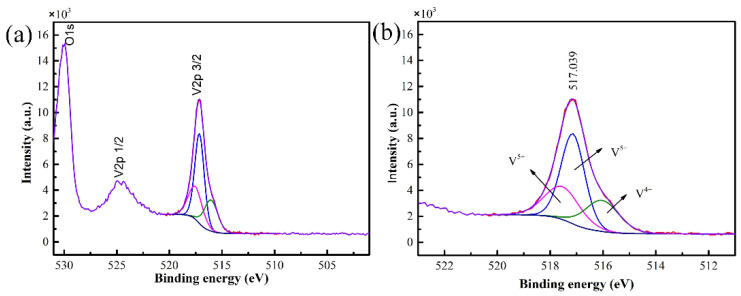
XPS spectra of the V_2_O_5_ film (**a**) and curve fitting of V2p_3/2_ spectra (**b**).

The valence state and composition information of the sample were obtained by XPS, and the results are shown in Figure 2. The binding energy for V2p_3/2_ peak was 517.039 eV, which corresponded to the characteristic binding energy of V^5+^, proving that the film prepared is V_2_O_5_ film [19].

### 3.2. Nonlinear Optical Properties

Then, the Z-scan method was taken to measure the nonlinear optical properties of the synthesized V_2_O_5_ thin film [20]. The laser source used in the Z-scan measurement had a wavelength of 1030 nm, the pulse duration was 1.2 ps, and the input pulse energy was 4.1 μJ. The laser beam was focused through a lens with a focal length of 200 mm, and the Rayleigh length Z_R_ and linear transmittance S were 1.15 mm and 0.2, respectively. For the open-aperture Z-scan mode, the normalized transmittance is described as [21]

(1) $$T_{N}\left(z,S=1\right)=\sum_{m=0}^{\infty }\frac{{\left[-q_{0}\right]}^{m}}{{\left(m+1\right)}^{3/2}}$$

where *q*_0_ is the free factor, which is defined as

$$q_{0}=\frac{\beta_{eff}I_{0}L_{eff}}{\left(1+z^{2}/z_{R}^{2}\right)}$$

*β_eff_* is the third order nonlinear absorption coefficient, *I*_0_ is the intensity of laser beam at focus position, and *L_eff_* is the effective thickness of the sample, which can be calculated by the following equation:

$$\;L_{eff}=\frac{1{-e}^{\left(-\alpha L\right)}}{\alpha }$$

where *L* is the thickness of the film, α is the linear absorption coefficient, which is defined as

$$\alpha =\frac{\log\left(\frac{1}{T}\right)}{d}$$

where *T* is the linear transmittance, and *d* is the thickness of the sample.

The third order nonlinear refractive coefficient *γ* can be given by the equation [22]:

(2) $$\gamma =\frac{\lambda \Delta \phi_{0}}{2\pi L_{eff}I_{0}}$$

(3) $$\Delta T_{p-v}=0.406{\left(1-S\right)}^{0.25}\Delta \phi_{0}$$

where Δ*ϕ*_0_ is the axial phase shift at the focus, and Δ*T_p−v_* is the difference value between the normalized transmittance peak and valley. Closed-aperture normalized transmittance can be simplified as [21]

(4) $$T\left(z\right)=1-\frac{4x\Delta \phi_{0}}{\left(x^{2}+9\right)\left(x^{2}+1\right)}$$

where x = Z/Z_R_, Z_R_ is the Rayleigh length.

**Figure 3:**
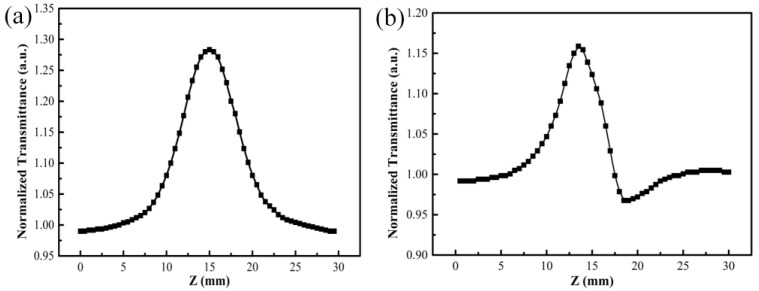
Open-aperture (**a**) and closed-aperture (**b**) Z-scan curves of V_2_O_5_ thin film with thickness of 90 nm.

In order to get the nonlinear coefficient of the V_2_O_5_ thin film, open-aperture and closed-aperture Z-scan measurements were conducted. The open-aperture Z-scan curve of the V_2_O_5_ film with thickness of 90 nm, as shown in Figure 3a, indicates that V_2_O_5_ thin film had a negative nonlinear absorption coefficient. It is obvious that with increasing irradiation intensity, the sample exhibited a saturable absorption. For the closed-aperture Z-scan characterization, as shown in Figure 3b, the normalized transmittance presented a peak profile followed with a valley, which was attributed to the self-defocusing process, indicating that the film had a negative nonlinear refractive coefficient. As the saturable absorption enhanced the nonlinear transmittance, the valley was well suppressed for closed-aperture Z-scan characterization. Equation (1) was used to fit the open-aperture curve to obtain the value of q_0_, and accordingly calculated the nonlinear absorption coefficient of −338 cm/GW. Moreover, the third-order nonlinear refractive coefficient was calculated to be −3.62 × 10^−12^ cm^2^/W by combining the Equation (2) with (3).

**Figure 4:**
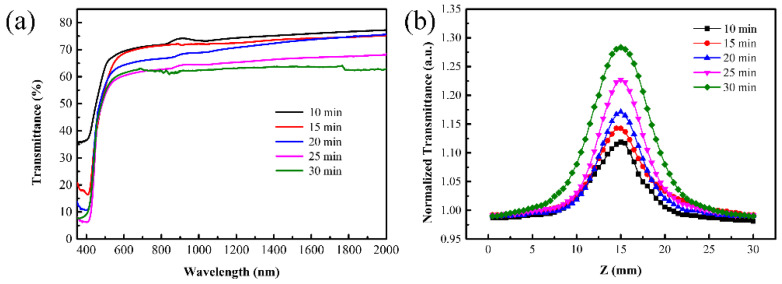
Transmittance spectra (**a**) and the open-aperture Z-scan curves (**b**) of the V_2_O_5_ film with different thicknesses.

Furthermore, the linear and nonlinear absorption properties of the V_2_O_5_ films with different thicknesses were investigated as shown in Figure 4. From the transmittance spectra of the films, as shown in Figure 4a, it can be derived that the transmittance of films decreased with increasing film thickness. The transmittance of the V_2_O_5_ films at the wavelength of 1030 nm was characterized to decrease from 73% to 62.5% when film thickness increased from 45 nm to 90 nm. The saturated absorption properties of V_2_O_5_ films with different thicknesses were characterized by the open-aperture Z-scan setup, as shown in Figure 4b. Based on the transmittance curves, the modulation depth of the V_2_O_5_ films with different thickness was calculated. As for the 45 nm V_2_O_5_ film, the modulation depth had a value of 13.8%. When the thickness was further increased, the modulation depth exhibited an apparent increase. The maximum modulation depth of V_2_O_5_ films obtained at a thickness of 90 nm was about 29.3%. Since the strength of the saturable absorption effect depended on the amount of the nonlinear materials, we see in Figure 4b a linear dependence of transmittance on the film thickness.

The relevant parameters of the nonlinear coefficient of the five V_2_O_5_ films with different thicknesses are listed in Table 1.

### 3.3. Phase Contrast Microscope

**Figure 5:**
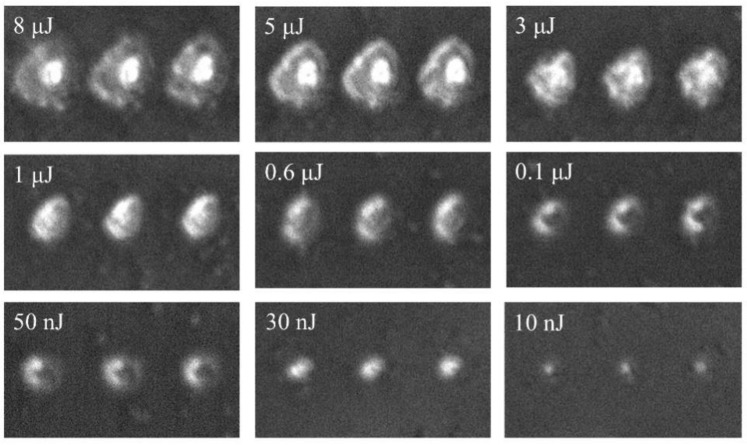
Phase contrast image of V_2_O_5_ after irradiated by ultrafast laser with different pulse energy. The focal beam waist is 697 nm.

The positive phase contrast microscope was used to characterize the morphology and index changes induced by the ultrafast pulsed laser. Figure 5 shows the phase-contrast images of V_2_O_5_ irradiated by the ultrafast laser with different pulse energies. The black region represents the positive refractive index change, while the white region represents the negative refractive index change. It can be seen that the refractive index of material experienced a negative change when the pulse energy was above 10 nJ, and the degree of the modification depended positively on the pulse energy. The size of laser-modified region increased with the increase in pulse energy. The threshold of refractive index modification under ultrafast laser irradiation was characterized to be about 863 mJ/cm^2^.

Local refractive index variation is the basis for optical devices such as waveguide and grating to produce optical function; therefore, it is very meaningful to study the refractive index modification of materials by ultrafast lasers. Since the focused ultrafast laser has an ultrahigh peak power intensity, which is enough to trigger the nonlinear absorption during laser–matter interaction, it results in local modification on material. The interaction between laser and matter is carried out through nonlinear effects within the focal length of the laser beam. After the sample was irradiated by ultrafast laser, most of the pulse energy was transferred to the material, which induced a series of refractive index modification-related processes, such as excitation and recombination. The natural phenomenon of laser irradiation weakens the strength of molecular bonds, which makes the material easier to expand and rarefact [23]. As the volume of the film expands, its density decreases, thus showing a negative refractive index change. This inference has been demonstrated in other papers; laser irradiation can form amorphous domains with lower density and, therefore, lower refractive index [24].

It is known from the previous Z-scan results that the prepared V_2_O_5_ had self-defocus characteristics and a negative third-order nonlinear refractive index, which implies a maximum of the index decrease at the center of the beam; these results are shown in Figure 5. It is the first time to observe nonvolatile refractive index changes at vanadium oxide. At present, the research on ultrafast laser three-dimensional processing material structure modification is a hot topic, and will lay the foundation for the design and production of thin film material modified devices. It is possible to design novel devices combining nonvolatile and volatile index changes. 

### 3.4. Raman Analysis

To analyze the structure changes of the film caused by different pulse energies, Raman spectra of different laser energy regions at room temperature were obtained by Confocal Micro-Raman spectrometer, which combines Raman spectrometer with microscopy, and could easily collect the spectra of a relatively small specific area of the film. Raman spectroscopy is very sensitive to the structure and the bond order of metal oxides, especially the stretching vibration mode of metal–oxygen bonds.

**Figure 6:**
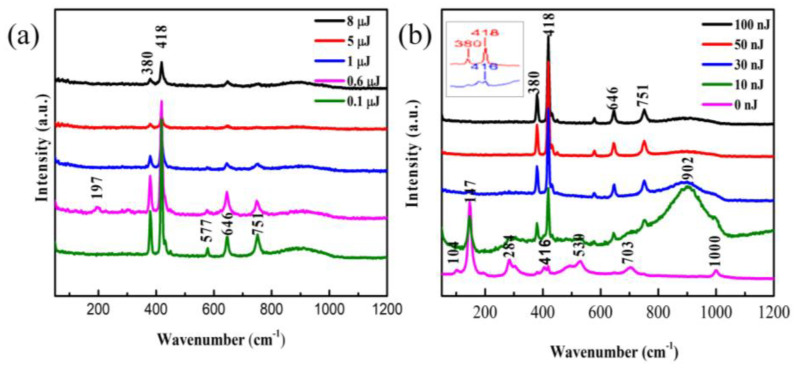
Raman spectra of V_2_O_5_ irradiated by high pulse energy (**a**) and low pulse energy (**b**). Inset shows the slightly shift of the peak at 416 cm^−1^.

As shown in Figure 6, the results show peaks at 104, 147, 197, 284, 380, 418, 416, 530, 646, 703, 751, 902, and 1000 cm^−1^. Compared with the unirradiated film (the curve corresponding to 0 nJ), the redshift of the Raman spectrum indicates the presence of the stress in the film. When there is a stress in the film, the bond length will change, therefore the vibration frequency of the bond will change, resulting in the shift of the Raman spectra. Hence, the peak located at 418 cm^−1^ is offset from the peak at 416 cm^−1^.

The untreated film exhibits Raman peaks centered at 104, 147, 284, 416, 530, 703, and 1000 cm^−1^ and most of these peaks were in good accordance with the literature reported for vibration mode of oriented crystalline V_2_O_5_ [25]. The presence of the Raman peak at 1000 cm^−1^ is evidence for the V_2_O_5_ film layered structure corresponding to stretching vibration mode of the terminal oxygen (V=O), which arises from an unshared oxygen [26]. The peak at 703 cm^−1^ is attributed to the doubly coordinated oxygen (V-O2-V) stretching vibration mode which results from corner-shared oxygens common to the two pyramids [25,27]. The peak at 646 cm^−1^ is assigned to the stretching vibration mode of the V2-O bond [27]. The peak at 577 cm^−1^ corresponds to VO_2_ [28]. The peak at 530 cm^−1^ is assigned to the triply coordinated oxygen (V-O3) stretching vibration mode which results from edged-shared oxygens in common to the three pyramids [27,29]. The peak at 416 cm^−1^ is assigned to a V-O-V stretching vibration mode, which can be attributed to the V_6_O_13_ [30]. The peak at 380 cm^−1^ is assigned to the monoclinic VO_2_ (M1) phase, which represents the vibration mode of V-O bond [31]. The peaks located at 284 cm^−1^ and 197 cm^−1^ are assigned to the bending vibration mode of the O3-V-O2 bond [25]. The predominant peak at 147 cm^−1^ is assigned to the skeleton-bent vibration of the V-O-V bond, which can be observed in crystalline V_2_O_5_ film [27,30]. The peak at 104 cm^−1^ is attributed to the external Ty mode [29]. The peaks at 751 cm^−1^ and near 902 cm^−1^ does not come from the V_2_O_5_, but from some of unidentified oxide of vanadium [28].

The Figure 6 shows that spectral peaks do not change significantly at irrdiated energy from 30 nJ to 8 μJ. Instead, there are a distinct changes in some peak strength with the changes of energy. That means a stabile state. However, at low irridated dose in Figure 6b the Raman spectra have big difference in compared with the initial vanadium oxide film. The Raman shifts indicates that the film is V_2_O_5_ [25]. After the irradiation with different pulse energies, the sample’s oxidation state changed, making the Raman peak position shift. Comparing the spectrum of untreated film with that of the laser irradiated film, we found that the low-frequency Raman peaks at 104, 147, 197, and 284 cm^−1^, representing the lattice V-V vibrations [29], disappeared after laser exposure. In other words, the disappearance of these peaks may be caused by the break of the V-V bonds. In addition, the Raman spectrum in the region with the pulse energy of 10 nJ looks similar to that of the unirradiated V_2_O_5_ film, possibly due to the effect that the modified region by such a low energy pulse is too small to be accurately selected by the Confocal Micro-Raman spectrometer. Therefore, the result may contain a large contribution from the unirradiated film. Between 800 and 1000 cm^−1^, there is a broad band with a center position of about 902 cm^−1^, and this feature is present in all other irradiated Raman spectra. The broad Raman bands were considered to be the superimposition of multiple merging modes. The peaks at high frequency (>800 cm^−1^) were assigned to V=O stretching vibration modes of distorted octahedra and distorted square-pyramids [32]. The new peaks at 751 and about 902 cm^−1^ did not correspond to any known vibrational mode of V_2_O_5_ and were explained to be associated with structural disorder [33]. In addition, the peak located at 902 cm^−1^ also appeared in other papers [30].

The Clausius–Mossotti equation told us that the refractive index of a solid is determined by the density of the constituent atoms and their corresponding polarizabilities [33]; laser irradiation will inevitably change the density of the irradiated region. In addition, the shift of the Raman spectra revealed that strain existed in the film, which led to a vibration of bonds length. Electron excitation is associated with the weakening and breaking of molecular bonds and formation of defects of color centers [23]. Each broken bond will produce a dangling bond or trigger a structure rearrangement process. Considering that the interaction between two oxygen atoms in the layered V_2_O_5_ film is relatively weak, the exsitence of oxygen vacancy and an oxygen dangling bond is also one of the important reasons for the refractive index modification.

The disappearance of initial peaks and the appearance of new peaks in the Raman spectrum indicated changes in the chemical composition of the irradiated region. Once the chemical composition changes, its physical properties including refractive index will inevitably change. In many optical glasses, the refractive index change is tightly associated with defect generation, bonds broken, and structure rearrangement [34]. Compared with optical glasses materials, V_2_O_5_ film possesses high nonlinear efficiency and relatively fragile, so changes in molecular structure occur more easily. In addition, the natural sapphire’s Raman peaked at 418 cm^−1^ [35]. Therefore, it is possible that the structure change may also have been caused by the decrease in the thickness of the film after laser irradiated.

## 4. Conclusions

In conclusion, we have prepared the V_2_O_5_ films with different thicknesses by using the radio frequency (RF) magnetron sputtering technique. The nonlinear absorption coefficient and the nonlinear refractive coefficient of the 90 nm V_2_O_5_ films were calculated to be −338 cm/GW and −3.62 × 10^−12^ cm^2^/W, respectively. The saturated absorption properties of the V_2_O_5_ films exhibits excellent controllability when varying the film thickness. The modulation depth of film can be tuned from 13.8% to 29.3%. Moreover, the refractive index modification by laser has been investigated and its physical mechanism has been discussed. The reasons for refractive index modification were the structure rearrangement (from V_2_O_5_ to V_6_O_13_), density variation, and defects. The progress of nanotechnology is largely dependent on the rapid development of ultrafast laser technology; the structural modification capability of ultrafast laser processing can reach nano-scale precision. Therefore, the research content of this paper is of great significance to optimize the performance of thin film optical devices.

## Figures and Tables

**Table 1:** Z-scan experimental data results.

Sample	T_1030_ (%)	Β_eff_ (cm/GW)	ΔT (%)
10 min	73.58	−141	13.8
15 min	72.05	−170	15
20 min	68.73	−204	18.1
25 min	64.43	−271	23.4
30 min	62.23	−338	29.3

## Data Availability

Not applicable.
